# Dental, dermatological and radiographic findings in a case of Gorlin-Goltz Syndrome: report and review

**DOI:** 10.11604/pamj.2017.27.96.12025

**Published:** 2017-06-07

**Authors:** Kumar Nilesh, Shivsagar Tewary, Sameer Zope, Jinesh Patel, Aaditee Vande

**Affiliations:** 1 Department of Oral & Maxillofacial Surgery School of Dental Sciences, KIMSDU, Karad, Maharashtra, India; 2 Department of Prosthodontics School of Dental Sciences, KIMSDU, Karad, Maharashtra, India; 3 Department of Periodontics School of Dental Sciences, KIMSDU, Karad, Maharashtra, India

**Keywords:** Nevoid basal cell carcinoma syndrome, kertocystic odontogenic tumor

## Abstract

Gorlin-Goltz syndrome (GGS) is a rare autosomal dominant disorder. The disease shows multiple organ involvement with variable clinical presentation. Thus a multidisciplinary approach is required for its prompt clinical diagnosis and management of this condition. This paper highlights a case of GGS presenting in a young male patient with cranial, facial, dermatological, dental and skeletal involvement. The diagnosis of the syndrome was based on its clinical presentation, radiological features and histopathological findings. A review of the diagnostic criteria is also presented.

## Introduction

Gorlin-Goltz syndrome (GGS) is an autosomal dominant disorder with extremely variable manifestations. It is a rare disease that is characterized by multisystem involvement; with a predisposition to cysts, neoplasms & other developmental abnormalities [1]. In 1894 Jarisch and White were the first to describe the essential features of this syndrome and termed it as nevoid basal cell carcinoma syndrome [2, 3]. Almost a century later, in 1960, Robert Gorlin and Robert Goltz delineated different clinical features of GGS in their paper on “multiple naevoid basal cell epithelioma, jaw cysts and bifid rib syndrome” [4]. The usual characteristic features of GGS include multiple keratocystic odontogenic jaw tumor, basal cell carcinoma, bifid ribs, palmar/planter pits and calcification of the falx cerebri. Along with these, numerous other features have been reported in literature, including developmental deformities like; maccrocephaly and cleft lip/palate, facial features like; frontal bossing and hypertelorism, skeletal abnormalities, and tumours including ovarian fibroma and medulloblastoma. With a background of the variable presenting features, Evans et al. enumerated major and minor criteria for diagnosis of the syndrome. This was later modified by Kimonis et al. in 1997, who stated that in order to establish diagnosis of GGS, at least two major and one minor or one major and three minor criteria must be present[5]. This paper reports the cranial, facial, dermatological, dental and skeletal manifestations of GGS in a young male patient. The syndrome was diagnosed based on its clinical presentation, radiological features and histopathological findings.

## Patient and observation

**Figure 1:**
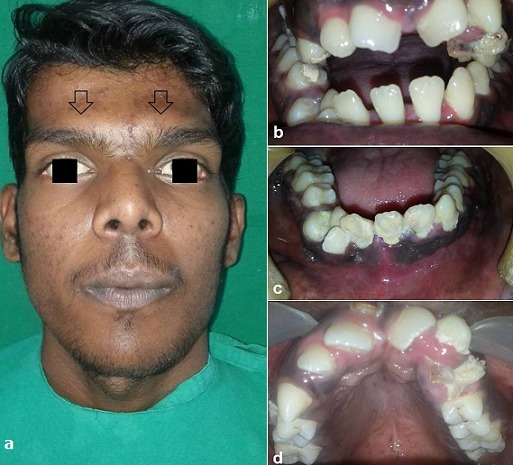
a) facial features of the patient; b) intra-oral findings showing anterior open bite; c) shallow buccal vestibule; d) high arched palate

**Figure 2:**
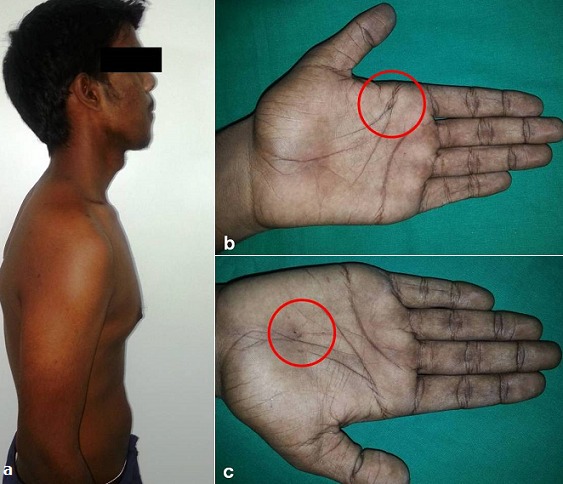
a) asymmetrical scapular position (Sprengel’s deformity); (b,c) palmar pitting

**Figure 3:**
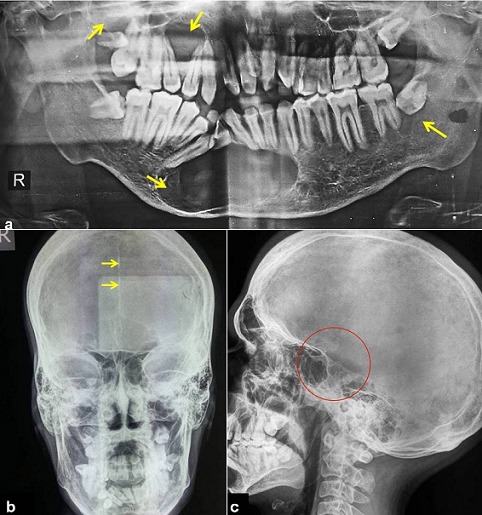
a) orthopantomogram showing multiple jaw cysts; b) posterio-anterior and lateral view of the skull showing calcification of falx cerebri; c) bridging of sella turcia

**Figure 4:**
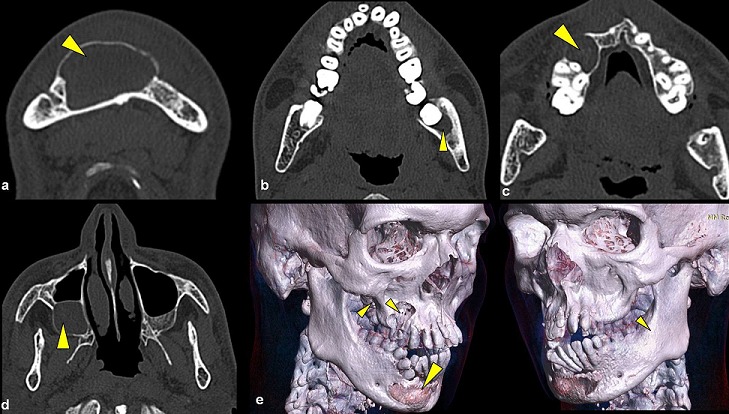
(a,b,c,d) transverse slides of CT, Formatted 3 dimensional images; e) showing multiple jaw cyst

**Figure 5:**
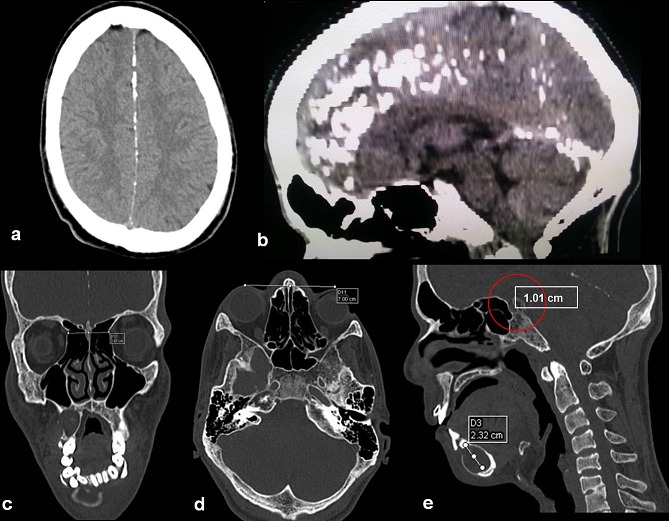
a) CT image showing multiple calcifications along the falx cerebrii; b) tentorium cerebelli; (c,d) linear measurements showing increased inter-canthal, orbit globe distance; e) narrowing of sella turcica

**Figure 6:**
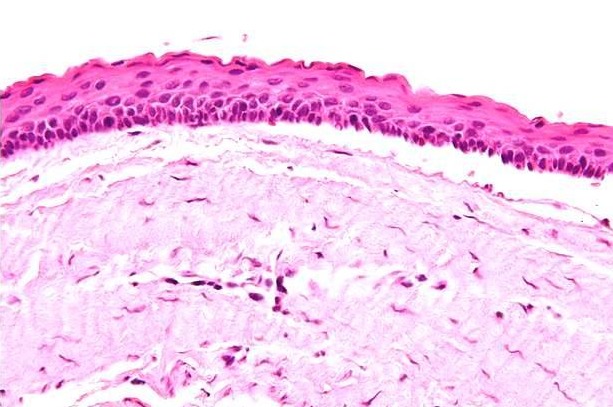
photomicrograph showing cyst lining (H & E stain; 10 X magnifications)

**Figure 7:**
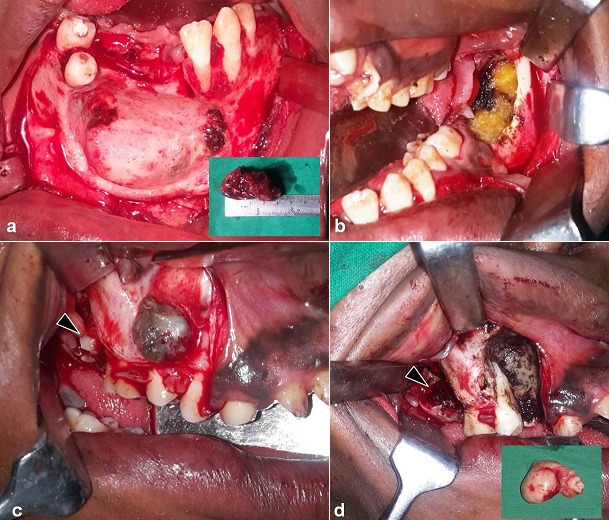
a) surgical removal of multiple KCOTs of jaw; enucleation of lesion; b) the bone cavity treated with carnoy’s solution; c) lesions of right maxilla; d: enucleated lesion

A 21 years old Indian male was referred to surgery clinic for evaluation of multiple radiolucent jaw lesions, which were seen on radiograph taken for routine dental treatment of the patient. The patient's family and medical history was non-contributory. The patient was perusing higher studies and on casual conversation his intelligence appeared normal. On facial examination mild frontal bossing, orbital hypertelorism, and incompetent lips were noted (Figure 1 a). Intraoral examination showed malaligned teeth with anterior open bite (Figure 1 b). There buccal vestibule was shallow with high arched palatal vault ( Figure 1 c, d). General physical examination showed mild imbalanced in the scapular position due to Sprengel´s deformity (Figure 2 a). Examination of skin on palmar surface showed punctiform brownish black depression on both hands suggestive of palmar pitting (Figure 2 b, c). Orthopantomogram of the patient revealed multiple radiolucent lesions involving both the jaw bones (Figure 3 a). A large radiolucency with well-defined margin was seen in anterior mandible. Two smaller radiolucent lesions associated with impacted left mandibular and right maxillary 3rd molar teeth along with periapical radiolucency between the root apices of maxillary right premolar were also noted. Posterio-anterior and lateral view of the skull taken to study skeletal discrepancies showed calcification of falx cerebri (Figure 3 b) and bridging of sella turcia (Figure 3 c). Chest, limb and cervical spine radiographs show no abnormality. Computed tomography (CT) of facial bones and cranium was advised for detailed radiological assessment. Sectional CT views showed a large well defined expansiolytic lesion with corticated margins of about 3.7 X 2.5 X 2.3 cm in size involving the mandible symphysis region. Lesion was seen growing along the length of the mandible, crossing symphysis mentii with impaction of associated canine teeth. Multiple similar lesions were noted in ramus of left mandible, alveolar process of maxilla on right side causing thinning of bone and abutting the ipsilateral maxillary sinus and right posterior maxilla extending into the maxillary sinus and showing impacted molar teeth within it (Figure 4). Incidentally multiple calcifications along the falx cerebrii and tentorium cerebelli were seen (Figure 5 a, b). Linear measurements on scan showed an increased in inter-canthal distance suggestive of hypertelorism and narrowing of sella turcica (Figure 5 c, d, e). The above dental, skeletal, cranial and dermatological features were suggestive of GGS with multiple jaw cysts. Incision biopsy of jaw cyst was carried under local anesthesia. Histopathological study of the specimen showed presence of six to eight cell layers thick parakeratinized stratified squamous cell epithelium suggestive of keratocystic odontogenic tumor (KCOT) (Figure 6). Enucleation of the jaw cysts along with chemical cauterization with carnoy's solution was performed under general anesthesia (Figure 7). Microscopic evaluation of the specimen lining was consistent for diagnosis of multiple KCOT.

## Discussion

Gorlin-Goltz syndrome (GGS) is a rare genetic disorder with extremely variable manifestations. The prevelance of the disease range from 1 in 57,000 to 1 in 2,56,000 in the general population [6]. It is caused by genetic mutation of the PTCH1 gene present on the long arm of chromosome 9. The mutation is transmitted in an autosomal dominant manner from parent to sibling. However 35 to 50% of GGS arise from spontaneous mutation, without any family history [7]. More than 225 mutations in the PTCH1 gene in Gorlin-Goltz syndrome have been identified so far. The PTCH1 gene functions in production of patched-1 protein that plays a role in cell growth and specialization. Mutation in this gene prevents the production or leads to the production of an abnormal protein that cannot effectively suppress cell growth and division. This results in an abonormal cellular proliferation resulting in formation of cyst/tumors that are characteristic of GGS. In the present report the patient had no family history and GGS was possibly due to spontaneous mutation. As with any syndrome, GGS is grouping of recognizable characteristics that occur together and have a common cause (i.e. mutation of PTCH1 gene). The clinical manifestations of GGS are highly variable with involvement across the organ systems. Evans et al. established the major and minor criteria for the diagnosis of the syndrome, which was later modified by Kimonis et al. in 1997 [5].

The major clinical criteria includes; more than two basal cell carcinoma or one basal cell carcinoma under the age of 20 years, KCOT, three or more cutaneous palmar or plantar pits, bifid/fused/splayed ribs and first degree relative with GGS. The minor creteri includes; macrocephaly, cleft lip or palate, frontal bossing, hypertelorism, skeletal abnormalities (Sprengel deformity, marked pectus deformity, marked syndactyly of the digits), bridging of the sella turcica, hemivertebrae, flame shaped lucencies of the hands or feet, ovarian fibroma and medulloblastoma. To establish a diagnosis of GGS, two major and one minor or one major and three minor criteria must be present (Table 1). Two of the major features; multiple KCOT, palmar pits and three minor criteria; frontal bossing, hypertelorism, and briging of sella turcia were positive for the present case. Other features incidentally noted were calcification of falx cerebrii and tentorium cerebelli. Intraoral findings of the case included malocclusion with anterior open bite, high arched palatal vault with obliteration buccal vestibule and incompetent lips. The principal clinical features of GGS comprise a triad of basal cell carcinoma (BCC), multiple KCOT and Skeletal anomalies, particularly of the thoracic cavity. Cutaneous BCC are the most frequent skin lesion of GGS. It is more frequent in white population (80%), compared to blacks (38%). Epidemiological studies suggest that sunlight, and particularly UV radiation, is a strong risk factor for the formation of BCC. It has recently been demonstrated that UV irradiation enhances BCC development in mice with PTCH gene mutation, thus confirming that BCC development in GGS patients is enhanced by UV irradiation. Presentation of BCC in GGS varies from a light to brown dark papules with a smooth surface and hard consistency to an ulceroproliferative lesion. The number of BCCs may vary from one to hundreds and is more common between puberty and 35 years of age. Sites mostly affected are thoracic and cervico-facial skin surfaces, periorbital areas, eyelids, nose, malar region, and upper lip. BCC was not a feature of the present reported case.

**Table 1:** criteria for diagnosis of GGS

Major criteria
More than two BCC or one BCC under the age of 20 years
KCOT of the jaw.
Three or more cutaneous palmar or plantar pits
Bifid, fused or markedly splayed ribs
First degree relative with GGS
Minor criteria
Macrocephaly
cleft lip or palate, frontal bossing, hypertelorism
Skeletal abnormalities: Sprengel deformity, marked syndactyly of the digits
Bridging of the sella turcica, hemivertebrae, flame shaped lucencies oF the hands or feet
Ovarian fibroma
Medulloblastoma

Another hallmark of GGS is occurrence of multiple KCOT of jaw [8]. Multiple KCOTs are the most consistent and representative signs of GGS in the first and second decades of life. Like in the present case, jaw cysts may be found as an incidental radiographic finding, during dental treatment. However it may clinically manifest as pain if the cyst is infected or cause symptoms such as swelling. KCOTs associated with GGS usually occur at an early age, usually in the first decade of life. Number of jaw cyst may range from a single lesion to as high as 30. In present case four cysts were discovered on orthopantomogram, occupying all the quadrants of jaw. KCOT are known to have a higher rate of recurrence. However the recurrence rate of KCOT in GGS is difficult to interpret because of the potential to develop multiple new cysts that occasionally may be confused with recurrence. Palmar and plantar pits are another major diagnostic clinical feature of GGS. These are multiple punctiform brownish black depression ranging from 2 to 3 mm in diameter and 1 to 3 mm in depth. Cutaneous pitting is caused by partial or complete absence of stratum corneum or dense keratin in sharply defined areas. It appears in 30%-65% of patients of GGS by the age of 10 years and in 85% of patients over the age of 20 years [9]. Various skeletal anomalies reported to be associated with GGS include high and broad forehead, frontal and parietal bossing. Facial features of broad nasal root are common and may be associated with ocular hypertelorism. The maxilla may be hypoplastic and there may be mandibular hyperplasia with variable prognathism. Other less common skeletal anomalies include a high arched palate, cleft palate and lip, malocclusions, multiple impactions of teeth.

## Conclusion

The syndrome shows high degree of penetrance with wide variability in its clinical manifestations. In view of multisystem involvement and an array of clinical and radiological presenting features of GGS, it is important to be aware of the major and minor diagnostic criteria of this disorder.

## Competing interests

The authors declare no competing interest.
